# The Effective Cognitive Assessment and Training Methods for COVID-19 Patients With Cognitive Impairment

**DOI:** 10.3389/fnagi.2021.827273

**Published:** 2022-01-11

**Authors:** Dong Wen, Jian Xu, Zhonglin Wu, Yijun Liu, Yanhong Zhou, Jingjing Li, Shaochang Wang, Xianlin Dong, M. Iqbal Saripan, Haiqing Song

**Affiliations:** ^1^Brain Computer Intelligence and Intelligent Health Institution, Institute of Artificial Intelligence, University of Science and Technology Beijing, Beijing, China; ^2^The Key Laboratory for Computer Virtual Technology and System Integration of Hebei Province, School of Information Science and Engineering, Yanshan University, Qinhuangdao, China; ^3^Department of Statistics, School of Science, Yanshan University, Qinhuangdao, China; ^4^Department of Computer Science and Technology, School of Mathematics and Information Science and Technology, Hebei Normal University of Science and Technology, Qinhuangdao, China; ^5^Department of Biomedical Engineering, Chengde Medical University, Chengde, China; ^6^Department of Computer and Communication Systems Engineering, Faculty of Engineering, Universiti Putra Malaysia, Serdang, Malaysia; ^7^Department of Neurology, Xuanwu Hospital of Capital Medical University, Beijing, China

**Keywords:** cognitive assessment and training, COVID-19 patients, cognitive impairment, brain-computer interface, virtual reality

## Introduction

Since late 2019, COVID-19 has been raging worldwide. Related studies have reported that many COVID-19 patients present cognitive sequelae (Ahmad and Rathore, 2020; Baschi et al., 2020; Gunasekaran et al., 2020; Heneka et al., 2020; Kieron et al., 2020; Koralnik and Tyler, 2020; Pinna et al., 2020; Vespignani et al., 2020; Woo et al., 2020; Taquet et al., 2021). However, the implementation of isolation measures greatly limits the traditional cognitive impairment assessment and treatment methods (Lara et al., 2020). Therefore, we need to explore better ways to assess and train cognitive impairment in patients with COVID-19.

The rapid development of a brain-computer interface (BCI), virtual reality (VR), and artificial intelligence has promoted the diagnosis and treatment of cognitive impairment in the direction of intellectual development. Studies have shown that BCI-VR technology can compensate for the limitations of BCI alone and provide new rehabilitation and assessment methods for patients with cognitive impairment, which has attracted increasing attention (Wen et al., 2018, 2020; Bauer and Andringa, 2020; Mancuso et al., 2020; Pinter et al., 2021).

This paper first discussed current cognitive impairment assessment and rehabilitation methods for patients with cognitive impairment. On this basis, we proposed a new comprehensive strategy for cognitive impairment assessment and rehabilitation based on BCI-VR. It will be a new approach for cognitive rehabilitation that fully integrates the accurate detection of BCI technology and the effective training of VR technology. Further, we discussed the advantages and challenges of BCI-VR in cognitive impairment assessment and rehabilitation training for COVID-19 patients in this current pandemic.

## Current Cognitive Impairment of COVID-19 Patients

Since the start of the COVID-19 pandemic, a growing number of studies have reported neurological impairment in COVID-19 patients (Ahmad and Rathore, 2020; Gunasekaran et al., 2020; Heneka et al., 2020; Kieron et al., 2020; Koralnik and Tyler, 2020; Pinna et al., 2020; Vespignani et al., 2020; Taquet et al., 2021). We found that stroke and cognitive impairment are the most common manifestations of neurological impairment in COVID-19 patients (Belani et al., 2020; Fara et al., 2020; Fatima et al., 2020; Haji Akhoundi et al., 2020; Heneka et al., 2020; Jain et al., 2020; Mahboob et al., 2020; Merkler et al., 2020; Rajdev et al., 2020; Sezgin et al., 2020; Taquet et al., 2021). Studies showed that the incidence of stroke in confirmed hospitalized COVID-19 patients ranged from 2.8 to 5.4%, the incidence of cognitive impairment was 26%, and patients with stroke often cause concurrent manifestations of cognitive impairment (Haji Akhoundi et al., 2020; Majidi et al., 2020; Oxley et al., 2020; Sun et al., 2020; Taquet et al., 2021). Other studies showed that patients with neurological impairment caused by COVID-19 tended to be younger (Ahmad and Rathore, 2020; Cavallieri et al., 2020; Oxley et al., 2020; Woo et al., 2020; Harrison et al., 2021).

In the current pandemic, both hospitals and patients are facing huge and severe challenges in the assessment and rehabilitation training of patients with cognitive impairment (Coetzer, 2020; Richardson et al., 2020). Studies showed, as the COVID-19 pandemic intensified, patients with cognitive impairment were limited in going out for rehabilitation training, which had many negative effects on the mental state of patients and his mental condition was deteriorating (Lara et al., 2020; Manca et al., 2020; Devita et al., 2021). Therefore, we need to explore better ways to assess and train cognitive impairment in patients with COVID-19.

## Current Evaluation Methods of Cognitive Impairment

Currently, common cognitive impairment methods include cognitive scale, neuroimaging technology and new wearable devices. In clinical practice, the most classic method of objective evaluation is the cognitive scale. The cognitive scale test achieves the evaluation effect by comparing the patient's test results with the scale indicators, include: Mini-Mental State Examination (MMSE) (Pangman et al., 2000), Montreal Cognitive Assessment (MoCA) (Nasreddine et al., 2019) and Activities of Daily Living (ADL) (Lopez Mongil, 2017), Auditory Verbal Learning Test (AVLT) (Stricker et al., 2021), Trail Marking Test (TMT) (Lunardini et al., 2020), etc. Recently, Burns et al. proposed a new hybrid scale—Free-COG, which could also be used to assess subjects' cognitive and executive functions (Burns et al., 2021). However, subjective factors of the testers reduced the accuracy of the results in the cognitive scale test.

The degree of cognitive impairment of patients is evaluated by observing the changes of brain structure through imaging (Knopman and Petersen, 2014). Among them, the commonly used neuroimaging techniques include: structural neuroimaging techniques (Zhang et al., 2019), functional neuroimaging techniques, positron emission tomography (PET), molecular imaging, and functional magnetic resonance imaging (fMRI) (Zhang et al., 2019; Xu et al., 2020). However, neuroimaging technology equipment is larger, and detection costs are higher, which significantly limits its application (Narayanan and Murray, 2016).

Relevant studies have introduced wearable devices into the assessment of cognitive impairment (Narayanan and Murray, 2016). Related study showed that wearable biosensor devices might be a viable tool to assess physiological changes in patients with AD, enabling remote and continuous monitoring of neurocognitive function in patients (Saif et al., 2019; Stavropoulos et al., 2020; Eggenberger et al., 2021). However, the evaluation indicators of the new wearable devices are uncertain, and there is no unified standard in use.

## Current Rehabilitation Methods of Cognitive Impairment

Currently, commonly used cognitive rehabilitation methods include medication-assisted, cognitive rehabilitation training, and home rehabilitation.

Medication-assisted therapy can inhibit the induction of cognitive impairment or other diseases (Rejdak and Grieb, 2020; Zhaojun and Miaser, 2020). The implementation of medication-assisted therapy is costly, and it also only serves as an adjustment role in the rehabilitation of cognitive impairment and may be accompanied by other side effects (Jin-xuan et al., 2020).

General cognitive rehabilitation training usually refers to systematic and targeted training depending on the patient's cognitive function under face-to-face guidance by the rehabilitation therapist. Studies showed it can improve or maintain patients' cognitive abilities related to daily task performance, so as to prevent or delay cognitive decline (Irazoki et al., 2020). But it requires the participation of both the therapist and the patient. And there are many limitations in the rehabilitation plan, such as time, personnel, and cost.

During the COVID-19 pandemic, traditional rehabilitation training is limited, researchers have suggested remote home rehabilitation for patients with cognitive impairment (Chang and Boudier-Revéret, 2020) and adopting some remote home rehabilitation measures (Richardson et al., 2020). Through literature analysis and comparison, it is found that home rehabilitation provides great convenience for both the therapists and patients, which can meet the needs of patients with cognitive impairment (Natta et al., 2015; Gately et al., 2019). However, long-term home rehabilitation reduces contact between patients with cognitive impairment and the outside world and has a certain impact on the patient's psychological state.

## BCI-VR Strategy for Evaluating and Rehabilitating COVID-19 Patients With Cognitive Impairment

It can be seen that there are many limitations in traditional cognitive impairment assessment and cognitive rehabilitation training during the COVID-19 pandemic. There is an urgent need for a novel and comprehensive strategy to overcome the shortcomings of traditional approaches. Thus, we propose a comprehensive rehabilitation strategy of BCI-VR, which combines the characteristics of accurate detection of BCI technology with the characteristics of effective training of VR technology and provide one-stop service for cognitive impairment assessment and cognitive rehabilitation training for COVID-19 patients. This strategy is described in detail below.

In BCI technology, EEG signals can be used to objectively and accurately detect the brain specificity of patients with cognitive impairment, which could be performed in the community or even at home (PoŽar et al., 2020; San-Juan et al., 2020; Pinter et al., 2021). VR technology provides an immersive environment for patients with cognitive impairment, improves patient participation and training effect (Snider et al., 2013; Appel et al., 2020; Bauer and Andringa, 2020; Mancuso et al., 2020; Thielbar et al., 2020). This makes the new rehabilitation strategy of BCI-VR proposed in this research more feasible.

**Figure 1:**
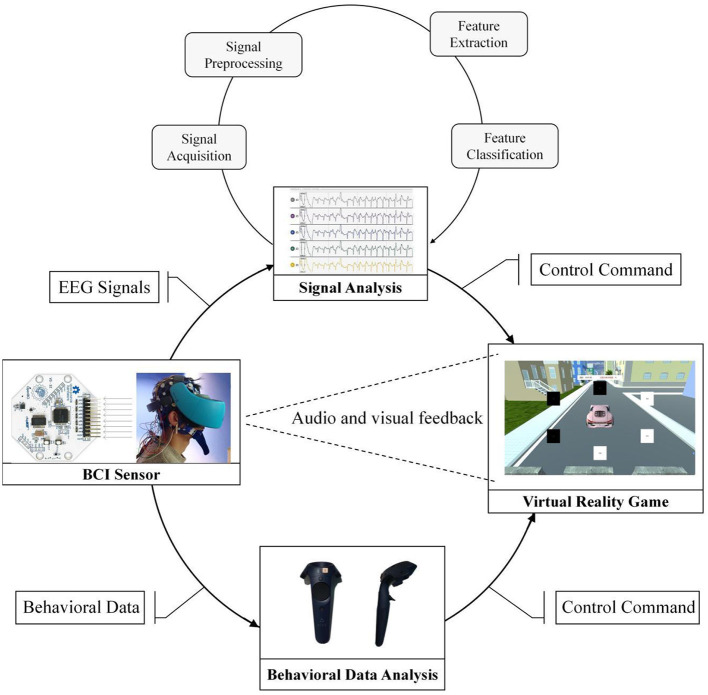
Diagram of the rehabilitation strategy based on BCI-VR.

The BCI-VR rehabilitation strategy proposed in this study provides guarantee for cognitive impairment assessment and cognitive rehabilitation training during the COVID-19 pandemic, which can meet the cognitive rehabilitation needs of patients at home. Moreover, the application of VR technology would greatly alleviate the negative psychological state and mental state of patients with cognitive impairment caused by blocking (Gao et al., 2020). In the implementation process (as shown in Figure 1), BCI-VR requires computers, EEG acquisition instruments and VR wearable devices, which are relatively light and common compared with medical devices in hospitals.

The BCI-VR strategy is specifically divided into a behavioral data analysis module and an EEG analysis module. During cognitive rehabilitation training in a VR environment, the EEG signals of patients with cognitive impairment are collected synchronously for offline or real-time online analysis (Taquet et al., 2021). Rehabilitation training data of patients with cognitive impairment in the VR environment will be recorded in the behavioral data analysis module. With the advancement of the rehabilitation training process, behavior data analysis can be intuitive to see its effect. For example, after a month of spatial navigation ability training, the first day of the spatial navigation ability will compare the 30 days of spatial navigation ability to test the result of rehabilitation training (Vespignani et al., 2020).

The EEG signals of cognitive impairment patients during rehabilitation training will be recorded and processed in the EEG analysis module. Continuous rehabilitation training will gradually show the characteristics of brain regions that constantly change. In patients, these changes can play an evaluation role.

Compared with traditional methods, BCI-VR has the following advantages: It reduces the need for patients with cognitive impairment to go out, and they can receive effective cognitive impairment assessment and rehabilitation at home; long-term home rehabilitation can relieve the mental state of patients with cognitive impairment, such as impatience and depression; it provides a low-cost cognitive rehabilitation strategy that uses relatively light and common equipment, which can be used in a wide range of applications.

## Discussion

More and more studies have been reported on the symptoms of cognitive impairment in COVID-19 patients (Baschi et al., 2020; Haji Akhoundi et al., 2020; Heneka et al., 2020; Jain et al., 2020). These patients need to undergo necessary cognitive impairment assessment and rehabilitation training after they are cured from COVID-19. Traditional cognitive impairment assessment and rehabilitation training have been greatly limited during the pandemic. BCI-VR provides a feasible method for patients in this situation. Recent studies have shown that the EEG signals of COVID-19 patients have certain characteristics (Sethi, 2020; Kubota et al., 2021). More and more researchers suggested that more attention should be paid to the EEG signals of patients during the epidemic (Haines et al., 2020). Perhaps BCI-VR may also monitor whether SARS-COV-2 virus reactivation occur while conducting cognitive rehabilitation training for COVID-19 patients after they are cured.

The application scenarios and implementation forms of the BCI-VR strategy are relatively flexible. It can be applied in rehabilitating various cognitive functions, such as memory, spatial cognition, or language perception, and multi-person interactive rehabilitation training, cross-scene interactive rehabilitation training, or cross-age rehabilitation training. Moreover, studies have shown that cognitive rehabilitation training with multi-person interaction in a VR environment has a better effect (Thielbar et al., 2020). Therefore, BCI-VR is better developed and applied in cognitive impairment rehabilitation.

BCI-VR in the assessment and rehabilitation of cognitive impairment is still in its early stages. In future research, BCI-VR will make great progress in integrating medical and industrial intelligence, which is not limited to cognitive impairment rehabilitation. However, the current optimized data fusion algorithm and feature extraction of high-dimensional data are still a bottleneck for BCI-VR development. In the following work, we will continue to solve the key BCI and VR technologies in monitoring, evaluating, and rehabilitating cognitive impairment.

## Conclusion

Through literature analysis and summary, we will find that more and more patients, including young people with COVID-19, exhibit signs of cognitive impairment. We analyze some popular traditional cognitive impairment assessment and rehabilitation methods and summarize their limitations during the current pandemic. Moreover, the proposed new comprehensive rehabilitation BCI-VR strategy and the cognitive impairment assessment and rehabilitation process of BCI-VR are expounded. The advantages of BCI-VR in cognitive impairment assessment and rehabilitation are discussed, and the development trend of this technology in the future is evaluated. However, the optimized data fusion algorithm and feature extraction of high-dimensional data are still the bottlenecks of BCI-VR development. Nevertheless, we expect that BCI-VR will soon play an important role in many fields, such as medical rehabilitation, providing more service support for humans.

## Author Contributions

DW, YZ, and HS contributed to conception and design of the study. JX, ZW, and YL searched the database. DW, JX, and JL performed the analysis of literature. JX, DW, and ZW wrote the first draft of the manuscript. YL and SW wrote sections of the manuscript. XD and MS revised this paper and analyzed the literature. All authors contributed to manuscript revision, read, and approved the submitted version.

## Funding

This work was supported in part by National Natural Science Foundation of China (Nos. 61876165 and 61503326) and National Key Research and Development Program of China (No. 2021YFF1200603).

## Conflict of Interest

The authors declare that the research was conducted in the absence of any commercial or financial relationships that could be construed as a potential conflict of interest.

## Publisher's Note

All claims expressed in this article are solely those of the authors and do not necessarily represent those of their affiliated organizations, or those of the publisher, the editors and the reviewers. Any product that may be evaluated in this article, or claim that may be made by its manufacturer, is not guaranteed or endorsed by the publisher.
